# Raised interleukin-10 is an indicator of poor outcome and enhanced systemic inflammation in patients with acute coronary syndrome

**DOI:** 10.1136/hrt.2007.119271

**Published:** 2007-08-09

**Authors:** A Mälarstig, P Eriksson, A Hamsten, B Lindahl, L Wallentin, A Siegbahn

**Affiliations:** 1 Department of Medical Sciences, Clinical Chemistry, Uppsala University, Sweden; 2 Atherosclerosis Research Unit, King Gustaf V Research Institute, Karolinska Institute, Sweden; 3 Department of Medical Sciences, Cardiology, Uppsala University, Sweden

## Abstract

**Objectives:**

To re-evaluate the relation between plasma interleukin-10 (IL-10) concentration at hospital admission and outcome and to investigate the impact of single nucleotide polymorphisms (SNP) in the IL-10 gene in patients with non-ST elevation acute coronary syndrome (ACS).

**Design:**

Determination of IL-10 plasma concentrations and genotyping of SNPs in the IL-10 gene in a prospective trial of patients with ACS and in a group of healthy controls.

**Patients:**

3179 patients in the Fragmin and fast revascularisation during InStability in Coronary artery disease II (FRISC II) trial and 393 healthy controls.

**Main outcome measures:**

Mortality and incidence of myocardial infarction (MI) at 12 months.

**Results:**

The median and interquartile ranges of IL-10 were 0.8 (0.5–1.0) pg/ml in healthy controls and 1.1 (0.7–1.9) pg/ml in patients (p<0.001). In patients, IL-10 predicted a crude risk increase of death/MI, with the highest risk observed in the fourth quartile (adjusted odds ratio 1.7 (95% confidence interval 1.2 to 2.3)). Adjustment for common risk indicators, including C-reactive protein and interleukin-6, weakened the association to a non-significant level. The 1170 CC genotype weakly predicted increased plasma concentrations of IL-10 in patients (p = 0.04) and in controls (p = 0.03), which was consistent with the modest association of this variant with coronary disease (p = 0.01).

**Conclusion:**

In contrast with some previous reports, we conclude that IL-10 reflects a proinflammatory state in patients with ACS and we therefore suggest that IL-10 is as effective a biomarker for the risk prediction of future cardiovascular events as other markers of systemic inflammation.

Acute coronary syndrome (ACS) is a clinical manifestation of coronary atherosclerosis and plaque disruption with superimposed thrombosis.^1^ ^2^ Inflammation is a significant component both in progression of atherosclerosis and in the acute thrombotic event, as evidenced—for example, by several studies associating proinflammatory markers with subsequent coronary events.^3^ ^4^ Accumulating evidence also speaks for a role of the immunoregulatory, anti-inflammatory cytokine interleukin-10 (IL-10) in the aetiology of ACS.^5^ ^6^ In vitro studies have demonstrated anticoagulant and anti-inflammatory properties of IL-10, such as suppression of tissue factor expression, cytokine production and interferon γ synthesis by activated monocytes/macrophages but also stimulatory effects such as B-cell growth and lipid accumulation in oxLDL (low density lipoprotein)-stimulated macrophages have been observed.^7^^–^11 In patients with ACS, low admission levels of IL-10 in serum samples have been associated with an increased risk of cardiovascular events and high IL-10 levels with a decreased risk.^12^^–^15 However, conflicting results have also been published.^16^ Therefore, the prognostic importance of IL-10 in patients with ACS is not well understood and a clinical consensus has not been reached.

Several reports describe associations between genetic variants in the IL-10 gene and increased secretion of IL-10 mRNA and protein.^17^^–^20 Of those, most studies have been restricted to the single nucleotide polymorphisms (SNP) at positions-1082 A>G, -819 C>T and -592 G>A in the IL-10 gene.

The primary aim of the present study was to compare plasma IL-10 concentrations in patients with non-ST elevation ACS with those in healthy subjects and to re-evaluate the relation between IL-10 levels at hospital admission and future risk of death and MI in patients. In addition, we investigated whether SNPs in the IL-10 gene were associated with coronary disease and with plasma concentrations of IL-10.

## METHODS

### Study participants

#### Non-ST elevation acute coronary syndrome

The patients in the present study participated in the Scandinavian multicentre trial FRagmin and fast revascularisation during InStability in Coronary artery disease-II (FRISC-II), which compared the benefits of an early invasive procedure over a non-invasive strategy and a prolonged treatment with a low-molecular weight heparin (dalteparin) versus placebo.^21^ ^22^ Patients were eligible if they had symptoms of ischaemia that could be verified by electrocardiography or increased biochemical markers and if the last period of chest pain had occurred within 48 hours from assignment to open-label dalteparin (study randomisation). Patients with contraindications to early revascularisation procedures or those included after official study closure were enrolled in the non-invasive arm only (n = 1032). In total, 3489 patients were included in the trial and followed regarding death and myocardial infarction (MI) for 12 months by an independent end point committee. The clinical end points were death, fatal or non-fatal MI or the composite of those within a follow-up time of 1 year.

### Healthy control subjects

Apparently healthy individuals (n = 393) who were similar in age and gender to the FRISC-II patients were recruited from the Swedish population registry.^23^ The healthy subjects were resident in southern, middle or northern parts of Sweden. Only those without a clinical history of cardiovascular disease or cardiovascular risk factors and with a normal electrocardiography and normal routine blood chemistry were eligible. Compared with the patient group, individuals who smoked were less frequent in the control group (14%). Written consent was obtained from all study participants. These studies, including the genetic substudy protocols, were approved by the regional ethics review board of Uppsala and conform with the principles outlined in the Declaration of Helsinki.

#### Early-onset myocardial infarction and matched healthy controls

The genetic associations observed in the FRISC-II cohort and healthy control group were followed up in the SCARF study, which included 362 survivors of MI and 381 age-matched and sex-matched healthy controls from the same county as the patients.^24^ The SCARF database and biobank were generated for studies of novel biochemical and molecular genetic markers of MI. All SCARF participants were below the age of 60 and blood sampling was performed 3 months after the index cardiac event. All subjects gave their informed consent to participation. The study was approved by the ethics committee at the Karolinska University Hospital.

### Laboratory analyses

Venous blood was drawn in citrated tubes (Vacutainer, Becton-Dickinson) at admission to the coronary care unit of the FRISC-II hospitals. Plasma was separated by centrifugation, aliquoted and stored at –70°C until analysis. The median time-lap between admission and blood sampling was 39 hours (interquartile range 27–55) hours. High sensitive IL-10 was measured using an ELISA technique with a lower detection limit of 0.5 pg/ml (R&D Systems, Minneapolis, MN, USA). The procedures for quantification of troponin-T (TnT), C-reactive protein (CRP), interleukin-6 (IL-6) and B-type natriuretic peptide (NT-proBNP) have been published.^25^ ^26^ Creatinine clearance was estimated by the Cockrauft-Gault equation.^27^ Plasma samples were available from 3179 FRISC-II participants and DNA from 2951 patients.

### SNP selection and genotyping

**Figure 1:**
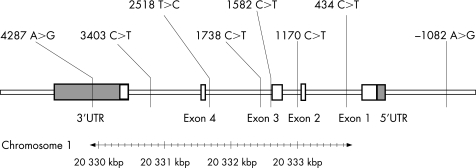
The IL-10 gene and the SNPs genotyped in the present study. The SNPs are indicated as position major>minor allele shift. Open boxes represent exons and shaded boxes represent untranslated regions.

Seven tag SNPs within and flanking the IL-10 gene were selected from the Seattle SNP database (fig 1).^28^ Genotyping was performed using the 12-plex GenomeLab SNPStream system (Beckman Coulter) or the homogeneous template directed dye terminator assay with fluorescence polarisation detection for individual SNPs.^29^ DNA was available from 2951 FRISC-II patients and all healthy controls. The overall genotype call rate was 95%, and the accuracy was 99.5% according to duplicate analysis of on average, 14% of all genotypes (3996/28 306). The IL-10 polymorphisms in all four subsets of patients and controls were distributed according to the Hardy-Weinberg equilibrium. The genotyping was performed by the SNP technology platform at Uppsala University (www.medsci.uu.se/molmed/snpgenotyping).

Genotyping for the IL-10 1170 C>T SNP in the replication study was performed using a pre-designed Taqman assay (C___8828803_1_) (Applied Biosystems, Foster City, CA, USA).

### Statistical analysis

Discrete data are presented as frequencies and percentages, and continuous variables as medians and interquartile ranges. Differences in levels of CRP and IL-10 between genotypes (or other categorical variables) were tested using the Kruskal-Wallis non-parametric test. Tests of independence for categorical factors were performed using the χ^2^ distribution with two-sided, exact p values. Logistic regression models were used to estimate the associations between categorised plasma levels of IL-10 and clinical end points. Regression model 1 adjusted for age, sex, body mass index, smoking status, hypertension, previous MI, diabetes, ST depression at entry, TnT >0.03 μg/l, NT-pro-BNP, creatinine clearance, use of aspirin, angiotensin converting enzyme (ACE)-inhibitor, β-blocker and statins. Regression model 2 also included CRP, as a continuous variable, and IL-6 plasma concentration above 5 ng/l. Statistics were calculated in SPSS for Windows XP 13.0.

## RESULTS

**Figure 2:**
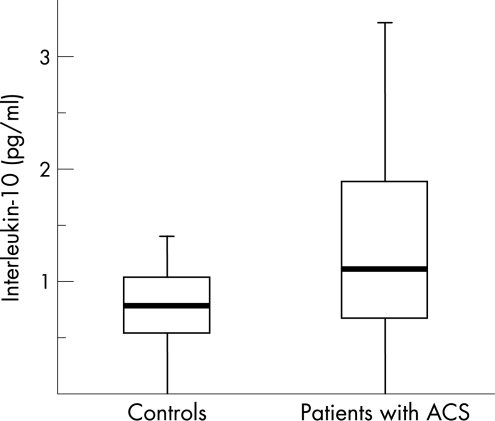
Box plot of IL-10 plasma concentrations in healthy controls and patients with acute coronary syndrome.

IL-10 plasma concentrations were significantly higher in the patient group than in the healthy controls (fig 2). The number of individuals with non-detectable IL-10 was 97 (24%) in the control group and 531 (17%) in patients.

**Figure 3:**
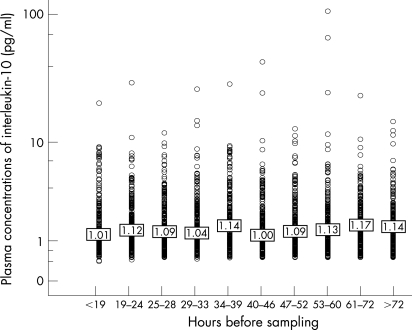
Hours between study randomisation and blood sampling.

To investigate the impact of different time-laps before sampling on IL-10 concentrations in patients, we compared IL-10 concentrations over deciles of hours before sampling. No statistically significant interaction between time before sampling and IL-10 concentration was observed (p = 0.72, Kruskal-Wallis) (fig 3). In addition, the proportion of patients with undetectable IL-10 was similar in patients with a short and long period of time before sampling (p = 0.21).

### Baseline characteristics by interleukin-10 median level

The FRISC-II patients were stratified according to the median IL-10 plasma concentration at admission. This analysis suggested considerable differences in the baseline characteristics of patients with IL-10 concentrations below and above median. Conventional risk factors were more frequent among patients with IL-10 concentrations above median, including elevation of CRP and IL-6 but excluding previous MI and hypertension. In contrast, smoking patients appeared to have a decreased IL-10 plasma secretion (table 1).

**Table 1:** Characteristics of FRISC-II patients according to IL-10 median

		Patients, IL-10 <1.1 pg/ml (n = 1602)	Patients, IL-10 ⩾1.1 pg/ml (n = 1577)	p Value
Median age (IQR)		66 (58–73)	68 (60–75)	<0.001
Men/women		1058/544	1118/459	0.003
Current smokers		466 (29%)	375 (23%)	<0.001
BMI		26.4 (24.3–28.9)	26.3 (24.3–28.9)	0.92
Diabetes mellitus		176 (11%)	234 (15%)	<0.001
Hypertension		505 (32%)	530 (34%)	0.21
Previous MI		419 (26%)	444 (28%)	0.21
ST depression at admission		711 (44%)	781 50%)	0.009
CRP (mg/l)		4.6 (2.5–9.2)	10.6 (4.2–32.3)	<0.001
TnT ⩾0.03 (μg/l)		1 005 (63%)	1 182 (75%)	<0.001
NT-pro-BNP ⩾529 (ng/l)		422 (43%)	577 (57%)	<0.001
Creatinine clearance <50		281 (18%)	381 (24%)	<0.001
IL-6 ⩾5 (ng/l)		171 (10.8%)	1 182 (44%)	<0.001

IQR (interquartile range), CRP (C-reactive protein), TnT (troponin-T), NT-pro-BNP B-type natriuretic peptide, IL-6 (interleukin-6).

### Interleukin-10 concentration at admission and risk of death and myocardial infarction

**Figure 4:**
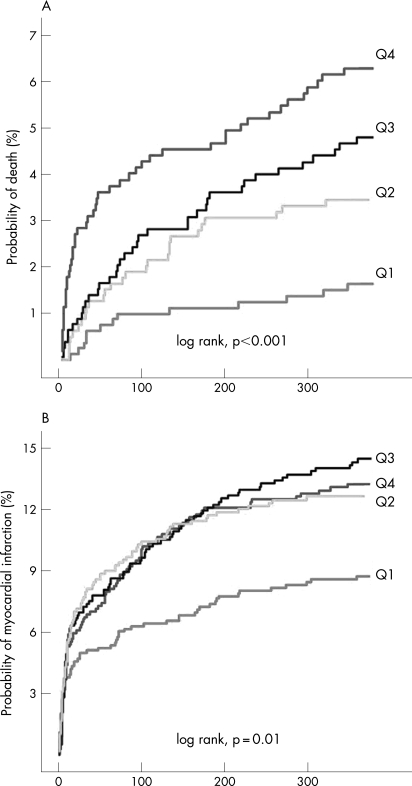
Probability of death and myocardial infarction (MI) according to IL-10 quartiles. (A) Kaplan-Meier estimate of the cumulative 12-month probability of death. (B) Kaplan-Meier estimate of the 12-month cumulative probability of MI.

The patients who were admitted with IL-10 concentrations in quartile 1 were at a significantly lower risk of subsequent death and MI compared with patients admitted with higher IL-10 concentrations (fig 4A and B). These trends were apparent in all treatment groups, as indicated by interaction tests between outcomes, IL-10 quartiles and treatment groups (data not shown).

Since IL-10 plasma concentrations were correlated with other risk factors, we investigated the predictive value of IL-10 quartiles using logistic regression models, which adjusted for conventional risk indicators (see statistics section). After adjustment, the risk of death in the highest IL-10 quartile remained (adjusted odds ratio 2.6; 95% CI, 1.3 to 5.1) (p = 0.007) (table 2). Additional adjustment for CRP and IL-6 (model 2) changed the mortality risk associated with high IL-10 to a non-significant level (adjusted odds ratio, 1.7; 95% confidence interval, 0.8 to 3.6) (p = 0.13) (table 2). The risk of MI associated with increased IL-10 was not significantly elevated after adjustment for the risk indicators in model 1 (table 2). Additional adjustment for CRP and IL-6 did not further alter this lack of association.

**Table 2:** Odds ratios for patient outcomes according to IL-10 level at admission

		IL-10 quartile	Univariate			Model 1		Model 2	
			Percentage (no. of events/total no.)	OR (95% CI)	p Value	Adj OR (95% CI)	p Value	Adj OR (95% CI)	p Value
Death		1	1.6% (13/805)	Reference		Reference		Reference	
		2	3.4% (27/797)	1.9 (0.9 to 3.8)	0.080	1.6 (0.8 to 3.2)	0.234	1.4 (0.7 to 3.0)	0.35
		3	4.7% (37/789)	2.7 (1.4 to 5.3)	0.004	1.8 (0.9 to 3.6)	0.105	1.5 (0.7 to 3.0)	0.30
		4	6.1% (48/788)	3.4 (1.8 to 6.5)	<0.001	2.6 (1.3 to 5.1)	0.007	1.7 (0.8 to 3.6)	0.13
MI		1	8.3% (67/805)	Reference		Reference		Reference	
		2	12.3% (98/797)	1.5 (1.1 to 2.2)	0.015	1.3 (0.9 to 1.9)	0.134	1.3 (0.9 to 1.9)	0.134
		3	13.3% (105/789)	1.7 (1.2 to 2.4)	0.002	1.3 (0.9 to 1.9)	0.107	1.4 (1.0 to 1.9)	0.066
		4	11.7% (92/788)	1.5 (1.0 to 2.1)	0.025	1.1 (0.8 to 1.6)	0.476	1.3 (0.9 to 1.9)	0.182
		1	9.4% (76/805)	Reference		Reference		Reference	
Composite of death/MI		2	14.3% (114/797)	1.5 (1.1 to 2.1)	0.011	1.3 (0.9 to 1.8)	0.130	1.3 (0.9 to 1.8)	0.172
		3	16.0% (126/789)	1.8 (1.3 to 2.5)	<0.001	1.4 (1.0 to 1.9)	0.07	1.3 (1.0 to 1.9)	0.095
		4	15.6% (123/788)	1.7 (1.2 to 2.3)	0.002	1.3 (0.9 to 1.8)	0.138	1.3 (0.9 to 1.8)	0.190

Model 1 adjusted for age, sex, BMI, previous MI, hypertension, diabetes, ST-depression at entry, dalteparin/placebo, TnT>0.03 μg/l, cholesterol >5.5 mM, smoking, creatinine clearance, NT-pro-BNP, use of aspirin, ACE-inhibitor, β-blocker and statins. Model 2 included also CRP and IL-6>5 ng/l.

### Association of the IL-10 1170 polymorphism with the acute coronary syndrome and premature myocardial infarction

The 1170 C allele was significantly more common in FRISC-II patients compared with the healthy control group (p = 0.01). None of the other SNPs were unequally distributed between FRISC-II patients and controls (table 3). To further investigate the relationship between 1170C>T and ACS we analysed this SNP in a cohort of patients with early-onset MI together with matched controls. However, the IL-10 1170 C>T SNP showed no association with premature MI (T allele: 22% vs 21%, p = 0.72).

**Table 3:** Single nucleotide polymorphisms in the IL-10 gene

			Minor allele frequency (%)		
SNP, alleles (major/minor)		rs number	Controls (n = 393)	FRISC-II patients (n = 2951)	p Value
−1082	A/G	rs1800896	49.2	49.6	0.84
434	C/T	rs2222202	49.3	49.0	0.86
1170	C/T	rs1518111	25.9	21.8	0.01
1582	C/T	rs1554286	21.7	19.2	0.07
1738	C/T	rs3024507	1.8	2.0	0.78
2518	T/C	rs3024509	6.0	6.4	0.60
3403	C/T	rs3024495	15.8	14.1	0.17
4287	A/G	rs3024498	27.0	26.9	0.95

*Tag SNP for IL-10 -819 C>T and -592 C>A.

Comparison of 1170 C>T allele frequencies between patients with and without a clinical end-point revealed no significant difference in the FRISC-II patient group (p = 0.09). After adjustment for other risk indicators (model 2), a moderate protective effect of the 1170 CT and TT genotypes was indicated (adjusted odds ratio, 0.7; 95% confidence interval, 0.6 to 1.0) (p = 0.036).

### Associations between the IL-10 1170 C>T polymorphism and plasma concentrations of interleukin-10 and C-reactive protein

**Figure 5:**
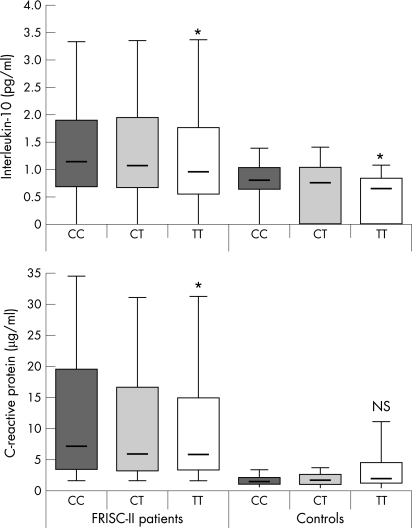
Plasma concentrations of interleukin-10 and C-reactive protein in patients and healthy controls according to 1170 C>T genotypes. The box plot represents medians, interquartile ranges and 10th–90th percentiles. *p<0.05.

The 1170 CC genotype was associated with approximately 20% higher IL-10 plasma concentrations in both FRISC-II patients and healthy controls (fig 5) (p = 0.04, p = 0.03 respectively). The IL-10 -1082 A>G SNP were unassociated as were the other polymorphisms genotyped in the present study. FRISC-II patients with the 1170 CC genotype also exhibited increased plasma levels of CRP (fig 5) (p = 0.02). A trend towards association with IL-10 plasma concentrations was observed in patients with premature MI in the SCARF study (median, interquartile range) (1170 CC: 1.5 (0.7–3.7), 1170 CT: 1.4 (0.8–3.4) 1170 TT: 0.8 (0.4–1.4) (mg/l) (p = 0.07).

## DISCUSSION

The present study shows that patients with ACS have higher IL-10 plasma concentrations than healthy controls and that elevation of IL-10 on admission was associated with a large number of common cardiovascular risk factors. In contrast to previous studies, a high concentration of IL-10 on admission was associated with a crude risk increase of death and MI. After adjustment for several conventional risk indicators, including markers for systemic inflammation, a clear association with patient outcome was no longer evident.

Our primary results poorly support previous smaller reports associating lower serum IL-10 levels with elevation of CRP, unstable angina or an unfavourable prognosis in patients with ACS.^12^^–^15 Of note, our study consisted of 3634 individuals with available plasma IL-10 determinations and is thus several-fold larger than any previous study on this topic. Therefore, we also had the possibility to investigate the distinct end-points of death and MI separately, which we demonstrated were relevant for the estimation of the predictive value of IL-10. Except for clinical end-points, the discordant results between our and previous studies may be attributable to differences in study design, biochemical methods, patient cohorts, medications, interventions and sampling time. Experimental studies have indicated that sampling time may considerably influence IL-10 measurements; IL-10 production was delayed in a study of patients measured before and after percutaneous coronary intervention and in vitro the production of IL-10 in lipopolysacharide-stimulated monocytes is delayed compared with that of interleukin-1β, interleukin-8 (IL-8) and tumour necrosis factor α (TNF- α).^30^ ^31^ As shown in figure 3, the influence of hours before sampling on plasma IL-10 concentrations was low for the different time intervals in our study. However, 98% of the FRISC-II patients were sampled later than 8 hours after study randomisation. Assuming that IL-10 concentrations are initially high for patients exhibiting lower IL-10 later in the acute course of events, a difference in the time lapsed before blood sampling may still explain the discrepancy between ours and a previous report on the prognostic value of IL-10 in ACS.^13^

Our findings suggest that a majority of patients with ACS present with considerably higher concentrations of IL-10 than those observed in healthy individuals. The magnitude of IL-10 elevation in patients was correlated with the extent of systemic proinflammatory activity, as evaluated by plasma concentrations of CRP and IL-6, which may be a reflection of coronary plaque rupture, thrombosis and cardiac damage. This observation leads to the notion that elevation of IL-10 is a consequence of the increased inflammatory activity seen in ACS, which also is related to long-term outcome.

The IL-10 gene is highly polymorphic and has been extensively studied in relation to expression phenotypes and association with human diseases. The 1170 C>T SNP is in complete allelic association with several other variants in the IL-10 gene, including the SNPs -592 C>A and -819 C>T, and was selected to tag these genetic variants. The A allele of -592 C>A and the T allele of -819 C>T have previously been linked to a lower expression IL-10 mRNA.^18^ Our results confirm that the 1170 C>T and linked polymorphisms, but not the -1082 G>A or other SNPs in our study, are genetic markers of in vivo IL-10 production. However, the genetic effect of these variants on IL-10 plasma concentrations was relatively small. The 1170 C>T C allele was significantly more common in FRISC-II patients compared to the age-matched and gender-matched healthy control group. The association was not further established in the replication study of young patients with early-onset MI, which may indicate that other co-factors are necessary for this variant to confer a risk increase. A weak association with recurrent events in the FRISC-II study was also observed after adjustment for cardiovascular risk factors. A recent report from the large PROSPER study demonstrated a significant association between a haplotype defined by the -592 C allele (in our study represented by the 1170 C allele) and future risk of coronary events, with an estimated hazard ratio of 1.2.^32^ In summary, our study and the PROSPER report suggest that the role of the IL-10 1170 C>T (and linked SNPs) in ACS is minor, which is also in accordance with the small effect of 1170 C>T on plasma concentrations of IL-10 and CRP.

The use of IL-10 as a biomarker for recurrent events in ACS is complicated by the complexity of both timing and magnitude of IL-10 secretion. Even so, our study showed that IL-10 plasma concentrations at admission well reflect the presence of common cardiovascular risk factors and can predict future cardiovascular events. The strong association between elevation of IL-10 and future events was significantly weakened only after adjustment for CRP and IL-6, which suggests that IL-10 holds similarly detailed prognostic information as other biomarkers of inflammation. Therefore, IL-10 may prove at least as valuable for risk prediction in ACS and can be considered as an alternative to other systemic inflammatory markers.
